# Histone deacetylase inhibitors exert anti-tumor effects on human adherent and stem-like glioma cells

**DOI:** 10.1186/s13148-018-0598-5

**Published:** 2019-01-17

**Authors:** Halina Was, Sylwia K. Krol, Dante Rotili, Antonello Mai, Bartosz Wojtas, Bozena Kaminska, Marta Maleszewska

**Affiliations:** 1 Laboratory of Molecular Neurobiology, Neurobiology Center, The Nencki Institute of Experimental Biology, 3 Pasteur Str, 02-093 Warsaw, Poland; 2 Laboratory of Molecular Oncology, Military Institute of Medicine, 128 Szaserow Str, 04-141 Warsaw, Poland; 3 Department of Drug Chemistry and Technologies, Sapienza University of Roma, P.le A. Moro 5, 00185 Rome, Italy; 4 Pasteur Institute, Cenci-Bolognetti Foundation, Sapienza University of Rome, 00185 Rome, Italy

**Keywords:** Glioblastoma, Histone deacetylase, HDAC inhibitors, Epigenetic drugs, Glioma stem cells, Cell proliferation

## Abstract

**Background:**

The diagnosis of glioblastoma (GBM), a most aggressive primary brain tumor with a median survival of 14.6 months, carries a dismal prognosis. GBMs are characterized by numerous genetic and epigenetic alterations, affecting patient survival and treatment response. Epigenetic mechanisms are deregulated in GBM as a result of aberrant expression/activity of epigenetic enzymes, including histone deacetylases (HDAC) which remove acetyl groups from histones regulating chromatin accessibility. Nevertheless, the impact of class/isoform-selective HDAC inhibitors (HDACi) on glioma cells, including glioma stem cells, had not been systematically determined.

**Results:**

Comprehensive analysis of the public TCGA dataset revealed the increased expression of *HDAC 1*, *2*, *3*, and *7* in malignant gliomas. Knockdown of HDAC 1 and 2 in human GBM cells significantly decreased cell proliferation. We tested the activity of 2 new and 3 previously described HDACi with different class/isoform selectivity on human GBM cells. All tested compounds exerted antiproliferative properties on glioma cells. However, the HDACi 1 and 4 blocked proliferation of glioblastoma cells leading to G2/M growth arrest without affecting astrocyte survival. Moreover, 1 and 4 at low micromolar concentrations displayed cytotoxic and antiproliferative effects on sphere cultures enriched in glioma stem cells.

**Conclusions:**

We identified two selective HDAC inhibitors that blocked proliferation of glioblastoma cells, but did not affect astrocyte survival. These new and highly effective inhibitors should be considered as promising candidates for further investigation in preclinical GBM models.

**Electronic supplementary material:**

The online version of this article (10.1186/s13148-018-0598-5) contains supplementary material, which is available to authorized users.

## Background

Glioblastoma (GBM, WHO grade IV) is the most common and aggressive primary tumor of the brain [1, 2]. GBM is characterized by rapid cell proliferation, high heterogeneity, extremely diffuse and infiltrative growth [3, 4], accompanied by extensive vascularization, and high resistance to standard therapies [5]. The median survival of patients with GBM is only 12.1–14.6 months from the time of diagnosis [5]. Conventional therapy for GBMs includes maximally safe surgical resection followed by radiation with concomitant temozolomide treatment, which prolongs survival, but is not curative. Therefore, the identification of new potential targets in GBM and the development of more effective therapies are urgently needed.

Recent findings from large-scale profiling including whole exome and RNA sequencing have revealed that both genetic and epigenetic mechanisms are significantly deregulated in glioma cells [6, 7]. In particular, alterations in sequence and/or expression of gene coding for HDACs may contribute to GBM pathogenesis and progression [2, 8, 9]. Various HDAC inhibitors (HDACi) have been tested in glioma, but none of them has passed to clinical practice so far, due to serious limitations, including toxicity and ineffectively low concentrations within the tumor [10]. Nevertheless, the impact of class/isoform-selective HDACi on glioma cells, including glioma stem cells, has not been systematically determined yet.

**Fig. 1:**
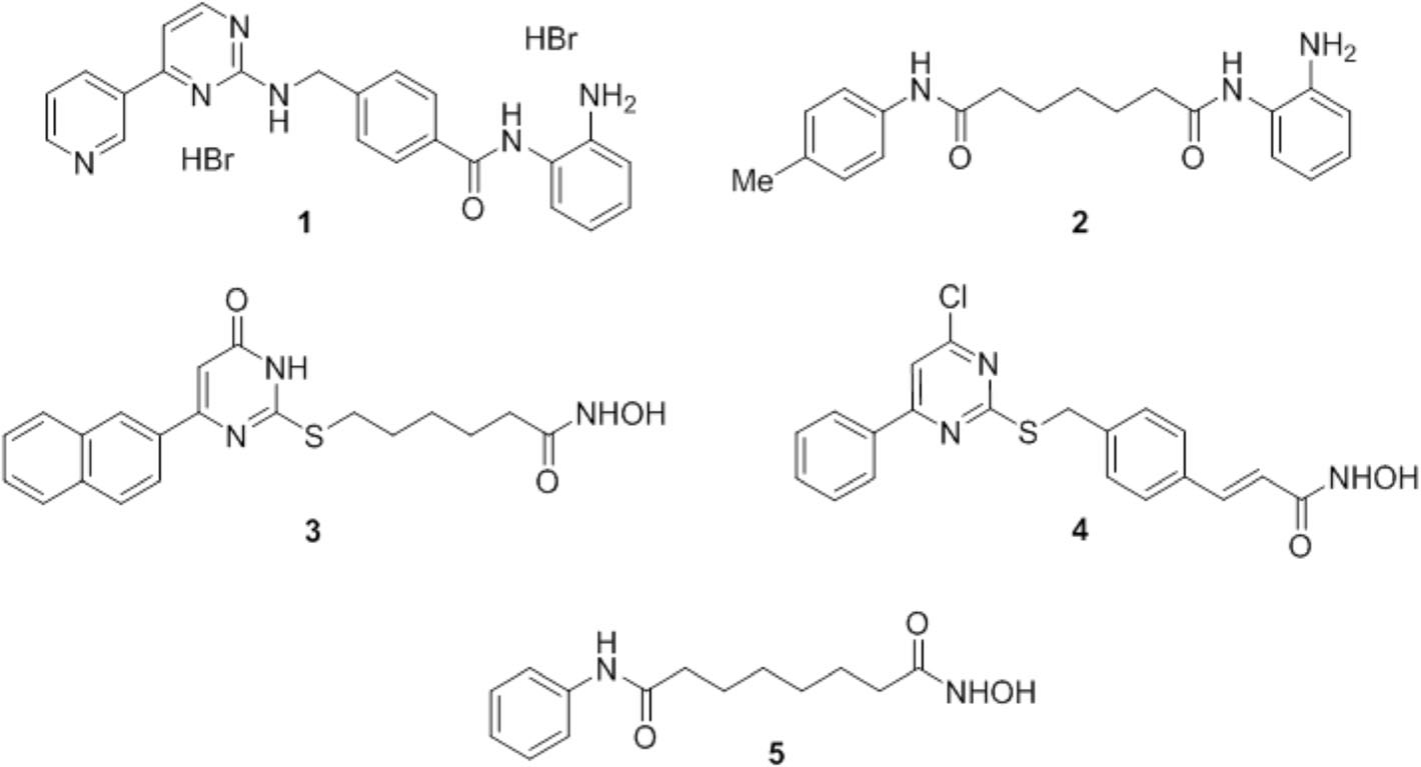
Structures of the HDACi **1**–**5** tested in the study: **1** mocetinostat, **2** compound 106, **3** MC1746, **4** MC2129, and **5** SAHA

In the present study, we investigated the short- and long-term effects of HDACi with different class/isoform selectivity on cultured human GBM cells, non-transformed glial cells, and glioma stem cell-enriched spheres. In particular, we tested mocetinostat (1, MGCD0103) [11] and compound 106 (2) [12] as prototypes of HDAC1/2 and HDAC3 selective inhibitors, respectively; MC1746 (3) and MC2129 (4), belonging to the class of the uracil-based hydroxyamides (UBHAs) [13–15] as new class I/IIb selective HDACi; and the FDA-approved SAHA (5, vorinostat) [11, 16] as reference pan-HDACi (Fig. 1). Relevant changes in 3 and 4 with respect to the classical UBHA scaffold [13–15] are the conversion of the C6-phenyl into C6-(2-naphthyl) moiety (3), a structural change known to improve the HDAC inhibitory potency in various series of hydroxamate HDACi [16], and the turning from C4-oxo to C4-chloropyrimidine (4), with the aromatization of the pyrimidine ring such as in 1 (Fig. 1) [9, 16].

## Results

### Selected HDACs are overexpressed in human glioblastomas

**Fig. 2:**
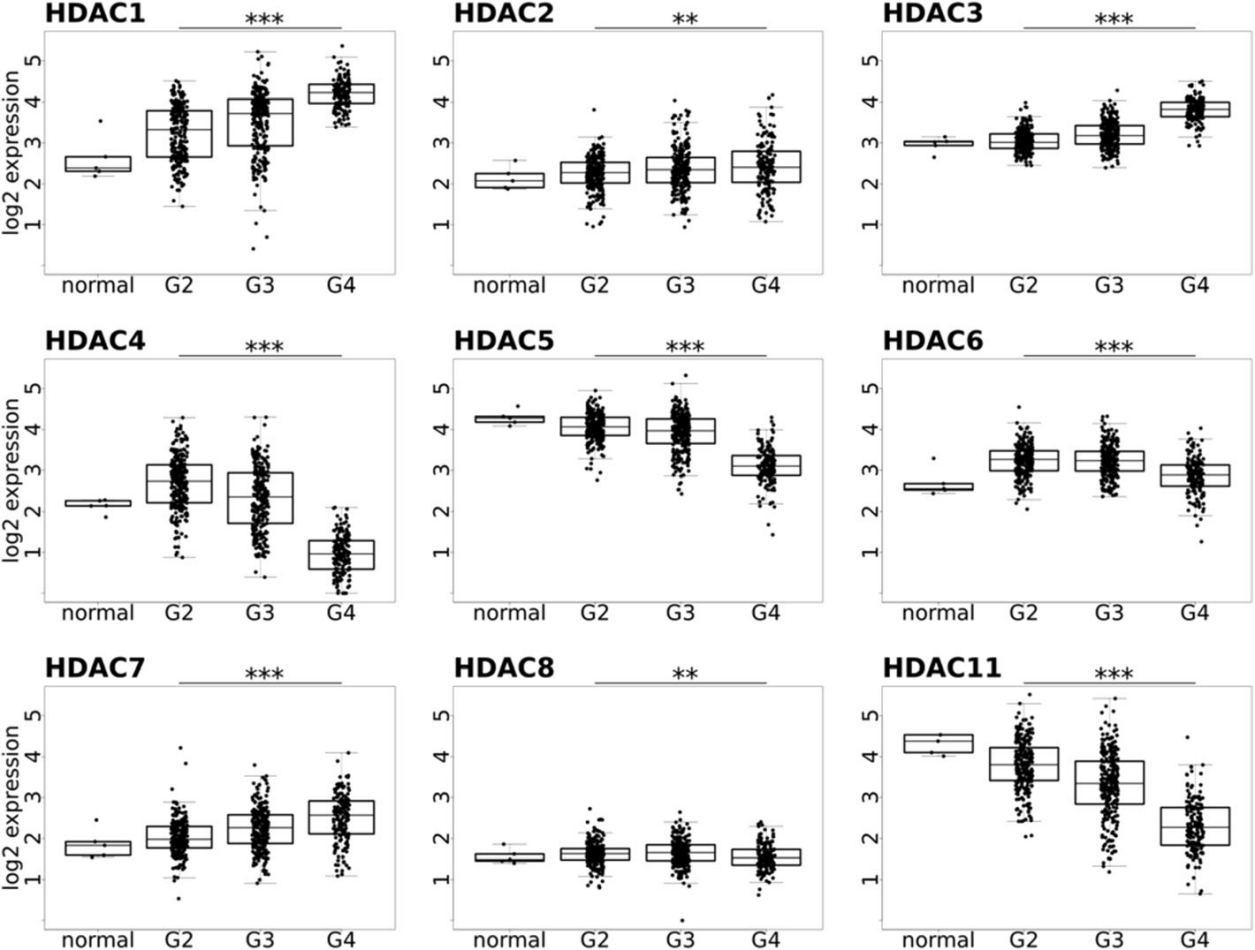
Expression of HDAC 1–8 and 11 in gliomas. Data were acquired from the TCGA repository as data level 3, FPKM values and include 5 normal brain samples (normal), 248 grade II (G2), 261 grade III (G3), and 160 grade IV (G4) tumor samples. Data were quantile normalized and log_2_ transformed. ANOVA test with no assumption of equal variance (Welch one-way test) was performed on all G2, G3, and G4 samples. Significant differences of means between G2, G3, and G4 were observed and denoted as significant after FDR corrections for multiple testing: *p value < 0.05, **p value < 0.01, ***p value < 0.001

The TCGA (The Cancer Genome Atlas) low-grade glioma (LGG) and GBM cohorts consist of 516 and 606 patients, respectively [7, 17]. By using RNA-sequencing data from the public TCGA database, the expression of the genes coding for HDAC 1–11 in five normal brains and a large group of gliomas of different WHO grades (GII-III-IV) was analyzed. In line with a previous report [18], the expression of HDAC 4, 5, 6, 8, and 11 was decreased in glioma tissues when compared to normal brains, particularly in GBMs (WHO grade GIV), so correlating inversely with malignancy grade, while the expression of HDAC 1–3 and HDAC 7 was significantly increased in high-grade gliomas (WHO grades GIII and GIV) (Fig. 2). HDAC 9 and 10 show no statistically significant differences in gene expression within TCGA cohort (data not shown).

### Effects of HDAC 1 and HDAC 2 knockdown on glioma cells

**Fig. 3:**
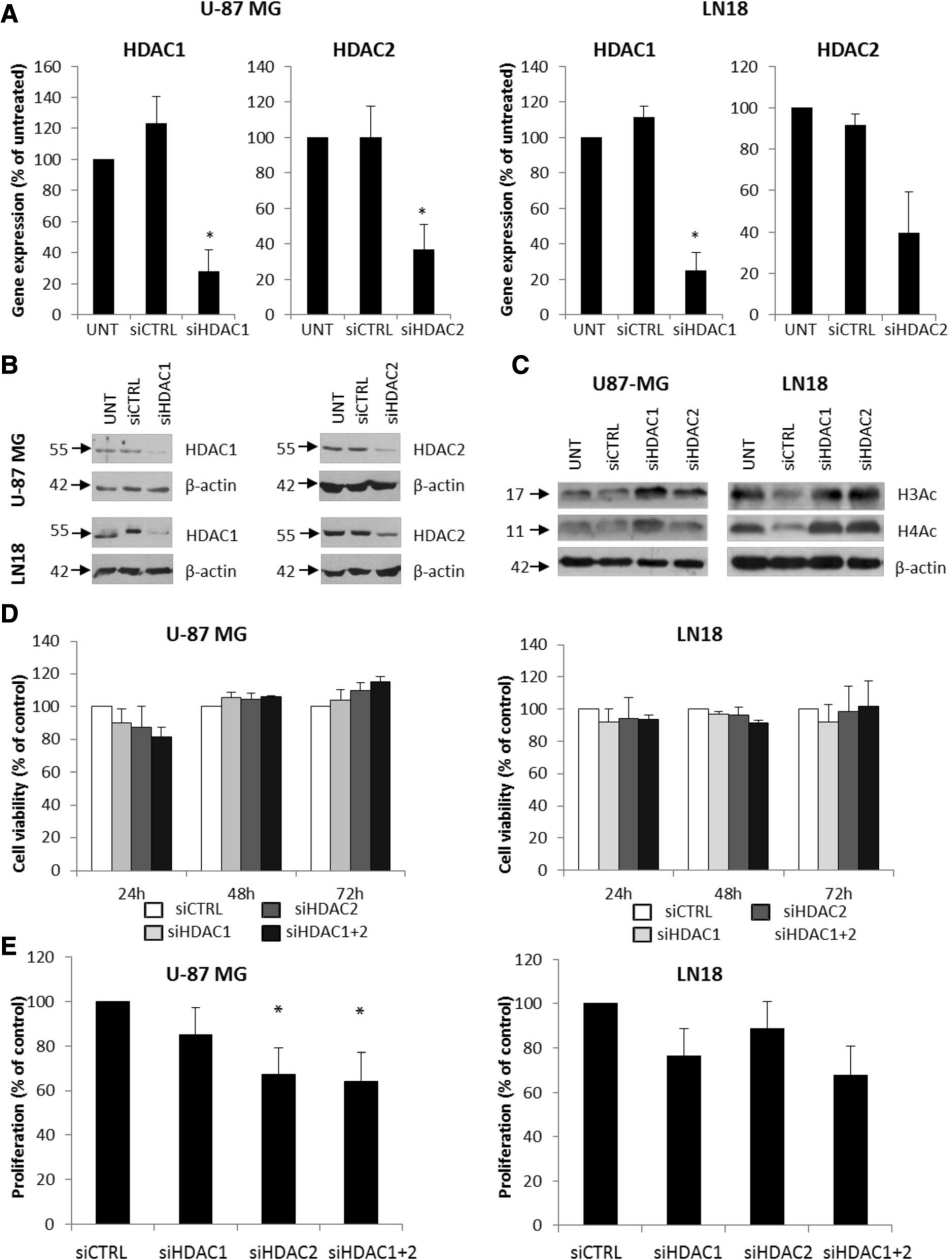
Knockdown of HDAC 1 and HDAC 2 results in reduced cell proliferation. **a** HDAC 1 and HDAC 2 expression was estimated by qRT-PCR in U-87 MG and LN18 cells after gene silencing using specific siRNAs. **b** Western blot analysis shows efficacy of HDAC 1 and HDAC 2 knockdown at protein level. **c** Western blot for acetylated histones H3 and H4 (H3Ac, H4Ac) in HDAC 1 and HDAC 2 depleted U-87MG and LN18 cells 48 h after siRNA transfection. **d** MTT metabolism test for cell viability 24, 48, and 72 h after transfection with HDAC 1 or/and HDAC 2 siRNAs or a control siRNA. **e** BrdU incorporation test for cell proliferation 48 h after knockdown of HDAC 1 or/and HDAC 2 in U-87MG and LN18 cells. The respective *p* values were calculated using type 2 two-tailed *t* test, and *p* < 0.05 was considered statistically significant. *p value < 0.05, *n* = 3

HDAC 1 and 2 are expressed in U-87 MG and LN18 glioblastoma cells. In order to determine the role of these HDACs in GBM, we knocked down their expression in U-87 MG and LN18 cells by using specific siRNA (ON-TARGET siRNA) and Viromer Blue as a transfecting agent. Transfectability of the labeled siRNA after treatment with viromer was estimated using fluorescence microscopy as 70–80% (not shown). In U-87 MG, the expression of *HDAC 1* at the mRNA level was reduced by 72.1% and *HDAC 2* by 75.0%, and in LN18 cells, the HDAC 1 and HDAC 2 expression was reduced by 63.1 and 60.3%, respectively (Fig. 3a) as determined by quantitative PCR (qPCR) and confirmed by western blot analysis at protein level (Fig. 3b and Additional file 1: Figure S1)). Concomitantly, increased levels of acetylated histones H3 and H4 were detected (Fig. 3c and Additional file 1: Figure S1). In both cell lines, the knockdown of either HDAC 1 or HDAC 2 or both did not significantly affect cell viability (MTT assay) (Fig. 3d), but inhibited glioma cell proliferation (Fig. 3e). Knockdown of HDAC 2 significantly reduced cell proliferation of U-87 MG cells and knockdown of HDAC 1 affected proliferation of LN18 cells. The effects of knockdown of both HDACs were not additive (Fig. 3e). Our results are in line with previous reports on cultured glioma cells [19, 20].

### Effects of HDACi on human glioma cells and normal human astrocytes

**Fig. 4:**
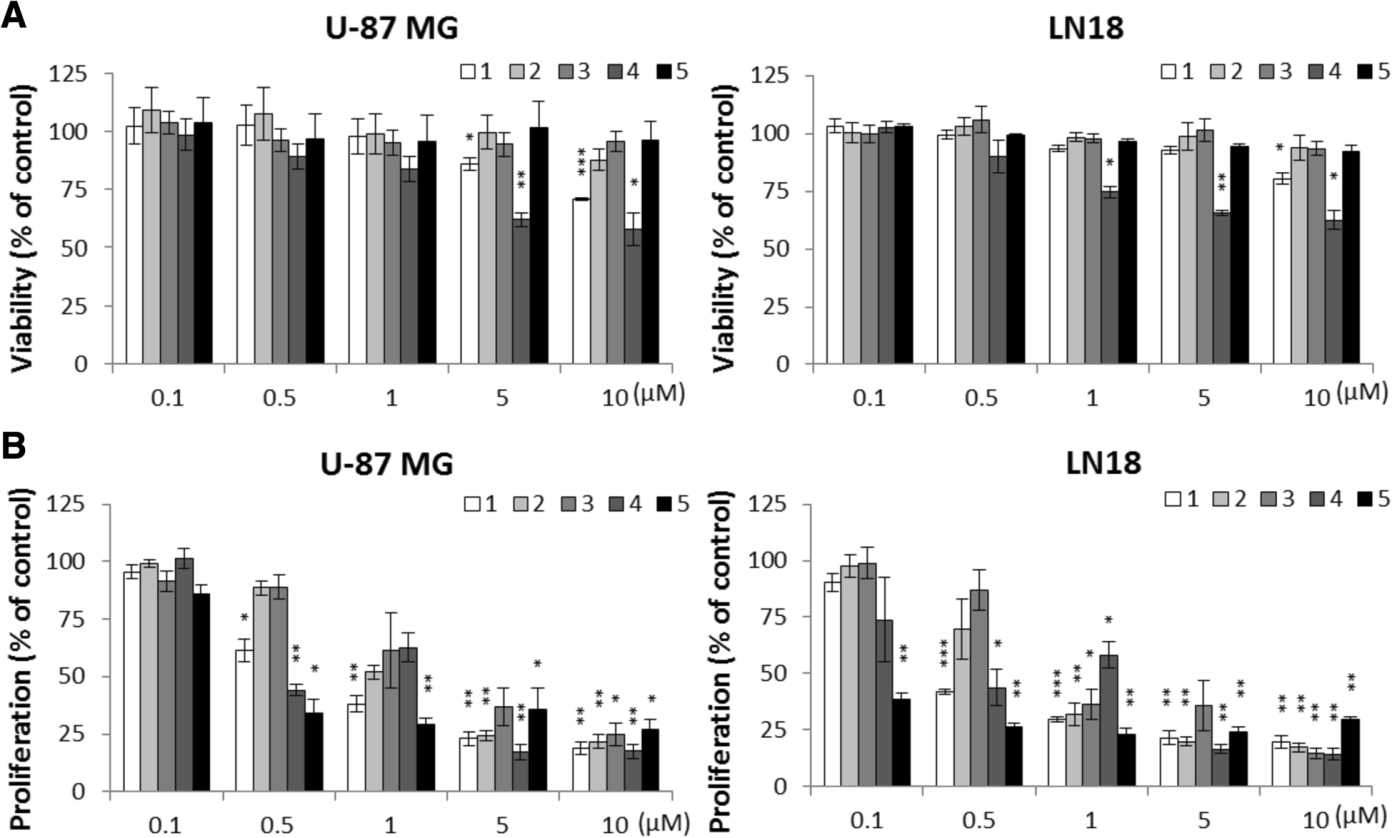
Effects of 1–5 on glioma cell viability and proliferation. **a** MTT metabolism test for cell viability of U-87 MG and LN18 cells after exposure to 1–5 for 24 h at indicated concentrations. Values for untreated cells were taken as 100%. **b** BrdU incorporation test for cell proliferation of U-87 MG and LN18 cells after exposure to 1–5 for 24 h at indicated concentrations. The respective *p* values were calculated using type 2 two-tailed *t* test followed by FDR corrections for multiple hypothesis testing and *p* < 0.05 was considered statistically significant (*n* = 3)

To overcome limitations of the siRNA-mediated knockdown, we studied the effects of the HDACi 1–5 on human glioma U-87 MG and LN18 cells. These compounds represent a set of inhibitors endowed with chemical heterogeneity and, with the exception of the pan-inhibitor 5, the capability to a certain extent discriminate among the various HDAC classes (1–4) and different isoforms within class I HDACs (1 and 2). The cytotoxicity of the investigated HDACi (delivered at concentration range 0.1–10 μM) was determined on glioma cells by MTT metabolism assay 24 h after treatment (Fig. 4a). Compounds 1 and 4 exerted the strongest cytotoxic effects and were effective in a dose-dependent manner in both cell lines at concentrations 1–10 μM. Other inhibitors did not show any significant effect on cell viability at the tested doses (Fig. 4a). On the other hand, HDACi 1, 2, 4, and 5 applied at concentrations from 5 to 10 μM for 24 h affected cell proliferation (determined by BrdU incorporation assay) in both cell lines (Fig. 4b). Again, compounds 1 and 4 exerted the strongest cytostatic effects at the concentration 5 and 10 μM and effectively reduced cell proliferation at concentration as low as 0.5 μM with results comparable to 5 (Fig. 4b). When tested at concentrations 1–10 μM for 24 h on normal human astrocytes (NHA), 1 and 5 did not reduce cell viability, which indicates a specificity towards tumor cells. In contrast to U-87 MG and LN18 glioma cells, NHA were also substantially not sensitive to 4 at 1 μM and 2 and 3 up to 5 μM (Additional file 2: Figure S2A). Compounds 3 and 4 had the effect on NHA morphology and increased the number of floating, dead cells. Cell shrinkage, detachment, and other morphological alterations suggest an occurrence of cell death in cell cultures (Additional file 2: Figure S2B). Treatment with the compounds 1, 2, and 5 resulted in increasing numbers of cells with enlarged and flatten morphology (Additional file 2: Figure S2B).

### HDACi exert mostly antiproliferative effects on human glioma cells

**Fig. 5:**
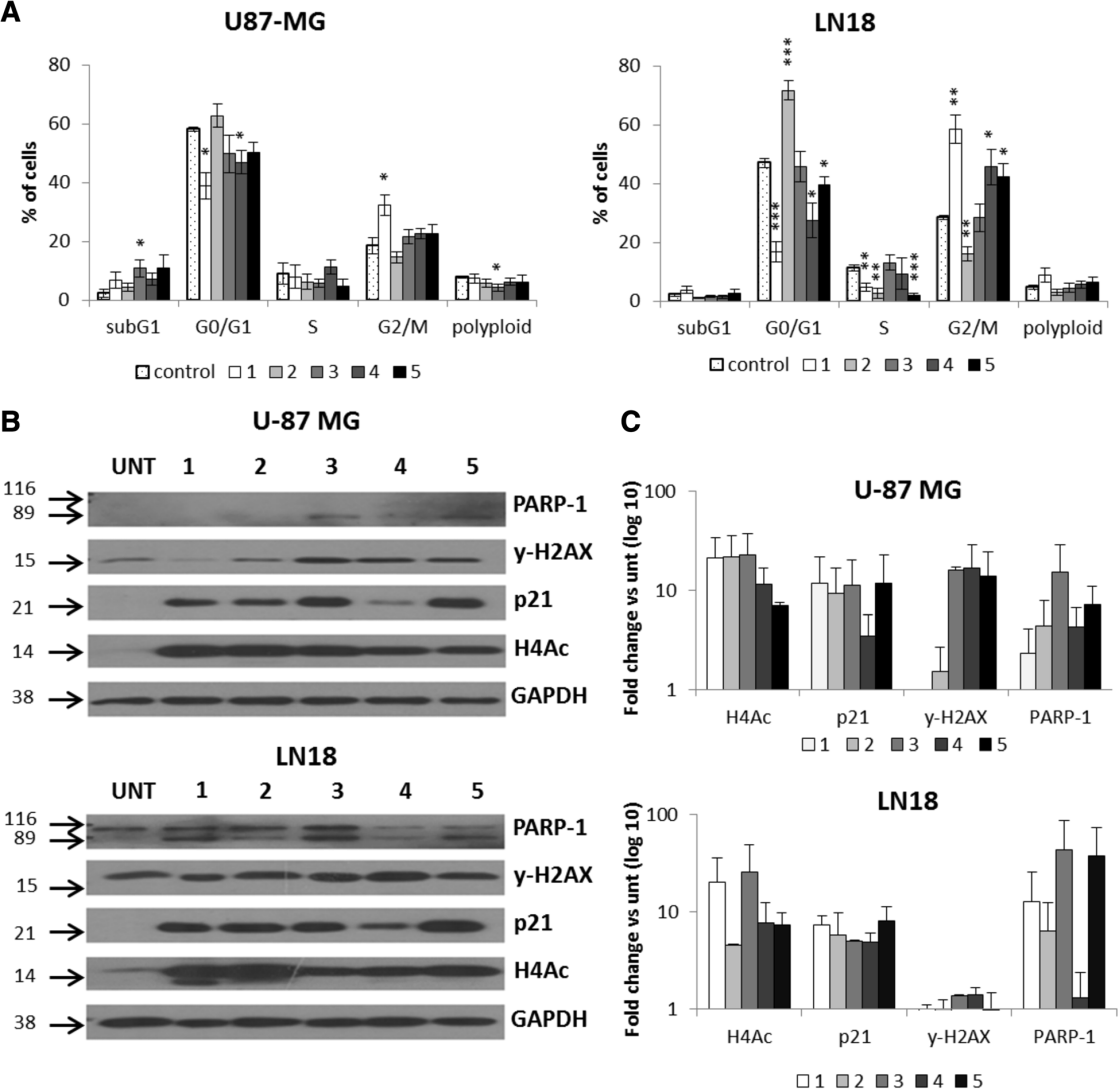
Antiproliferative effects of 1–5 on glioma cells. **a** The effects of compounds 1–5 on cell cycle in glioma cells were determined by propidium iodide (PI) staining and flow cytometry. Quantification of three experiments is presented. The respective *p* values were calculated using type 2 two-tailed *t* test, and *p* < 0.05 was considered statistically significant: *p value < 0.05, **p value < 0.01, ***p value < 0.001. **b** Total protein extracts were collected from U-87 MG and LN18 cells exposed to HDACi. Representative immunoblot shows results of western blot analysis of PARP-1 cleavage, γ-H2AX, p21, and H4Ac levels in U-87 MG and LN18 cells after exposure to 5 μM 1–3, 5, and 1 μM 4. **c** Densitometry analysis of western blot of PARP-1 cleavage, γ-H2AX, p21, and H4Ac levels in U-87 MG and LN18 cells after exposure to 5 μM 1–3, 5, and 1 μM 4 (*n* = 2)

In order to better understand the effects of 1–5 in U-87 MG and LN18 glioma cells, the distribution of cells in different phases of the cell cycle was measured by using propidium iodide (PI) staining and flow cytometry (Fig. 5a). Both cell lines were treated with 1–5 at 5 μM (except 4, tested at 1 μM since at 5 μM, we already observed a dramatic decrease in glioma cells viability) for 24 h. Compounds 1 and 4, more efficiently than 5, caused significant accumulation of LN18 cells in the G2/M phase with the concomitant reduction of cells in the G0/G1 and S phases, while 2 induced the G0/G1 phase arrest and decreased percentage of cells in G2/M and S phases (Fig. 5a). The effects of 1 and, to a lesser extent, of 4 and 5 were similar in U-87 MG cells (Fig. 5a).

In both cell lines, the tested compounds strongly increased the levels of acetyl-H4 and induced the expression of the cell cycle inhibitor p21^WAF1^, leading to G2/M or G0/G1 cell cycle phase arrest and programmed cell death as demonstrated by western blot analysis (Fig. 5b, c). The expression of γ-H2AX, a component of the DNA damage response (DDR) pathway, was affected in U-87 MG cells, when treated with HDACi 3, 4, and 5, while the levels of cleaved PARP-1, a substrate of activated caspase 3, increased mainly in LN18 cells, confirming a programmed cell death. It was probably non-apoptotic death, as the subG1 population marking DNA fragmentation was not detected (Fig. 5a). The different cellular responses to 1–5 in U-87 MG and LN18 cells were also confirmed by the effects on cell morphology (Additional file 3: Figure S3). Indeed, while the treatment of U-87 MG cells with 1, 2, and 4 resulted in an increased number of cells with an enlarged and flattened shape, in LN18 cells, the treatment with 1, 2, 4, and 5 augmented the number of rounded, dead cells (Additional file 4: Figure S4). Such morphological alterations are associated with growth arrest or non-apoptotic programmed cell death of malignant glioma cells, respectively [21].

### HDACi treatment induces long-lasting effects in human glioma cells

**Fig. 6:**
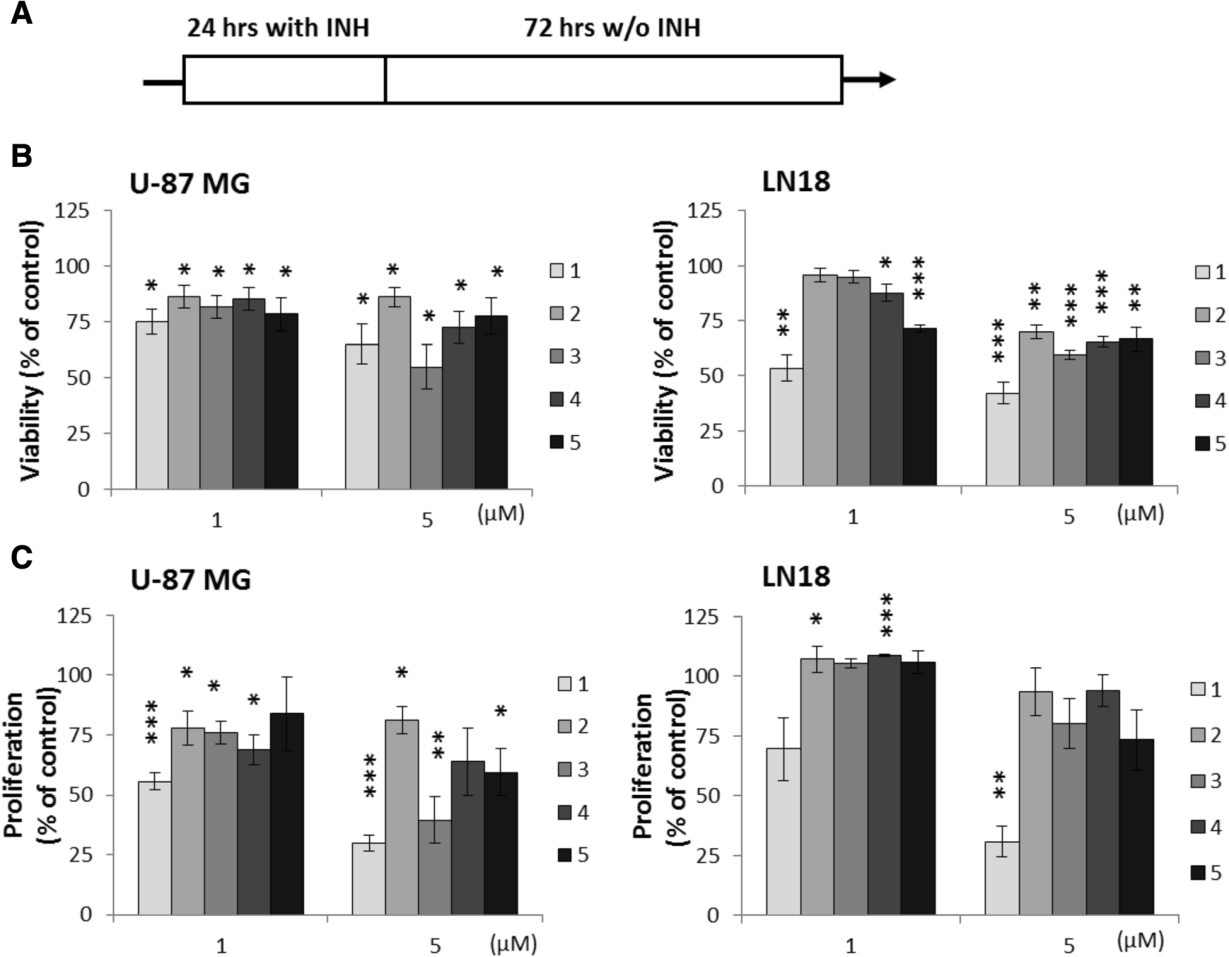
Long-term effects of 1–5 on glioma cell viability and proliferation. **a** Scheme of the experiment—the cells were exposed to 1–5 for 24 h, then HDACi were washed out and cells were cultured for another 72 h. **b** MTT metabolism test for cell viability of U-87 MG and LN18 cells after exposure to 1–5 at indicated concentrations. **c** BrdU incorporation test for cell proliferation of glioma cell after exposure to 1–5 at indicated concentrations. Values for untreated cells were taken as 100%. The respective *p* values were calculated using type 2 two-tailed *t* test, and *p* < 0.05 was considered statistically significant. *n* = 3, *p value < 0.05, **p value < 0.01, ***p value < 0.001

In order to investigate whether the treatment with 1–5 induces long-term changes in glioma cells, compounds were washed out after treatment of U-87 MG and LN18 cells with 1–5 for 24 h, and the cells were cultured for additional 72 h in a HDACi-free medium (Fig. 6a). All tested HDACi reduced the viability of U-87 MG and LN18 cells at concentrations 1 and 5 μM, although for compounds 2 and 3 at 1 μM, the results were not significant in case of LN18. At 5 μM, the cytotoxic effects were more pronounced in LN18 cells (Fig. 6b), with 1 being the most potent and 3 and 4 displaying higher effect than 5 at the highest tested dose in both cell lines. Antiproliferative effects induced by 1–5 were more evident in U-87 MG cells, with 1 and, to a lesser extent, 3 and 4 being the most effective (Fig. 6c). Different effects on cell morphology were also observed in those long-term cultures, where most compounds (except 2) increased the number of floating, dying cells in LN18 cultures, while in U-87 MG cultures, 1 induced profound morphological changes: flattened morphology with protracted extensions, comparable to those produced by 5 (Additional file 4: Figure S4).

### Effects of HDACi on human glioma cancer stem cells

Cancer stem cells (CSCs) are a rare subpopulation of cancer cells that are capable of self-renewal and are more resistant to anti-tumor therapeutics than bulk cells [22]. Therefore, eliminating CSCs is considered a new, attractive anticancer therapeutic strategy. We have previously implemented a protocol to cultivate LN18 sphere cultures enriched in glioma CSCs which expressed higher levels of the pluripotency markers: *NANOG*, *POU5F1*, *SOX2*, and *CD133* as compared to the parental/adherent tumor cells [23–25]. LN18 cells were seeded into non-adherent dishes, cultured in a serum-free medium, and supplemented with basic FGF and EGF for 6 days when they formed spheres of the size ≥ 150 μm [24]. We employed such protocol to grow LN18 spheres, and 6-day sphere cultures were treated with 1–5 for 24 h [22–25].

**Fig. 7:**
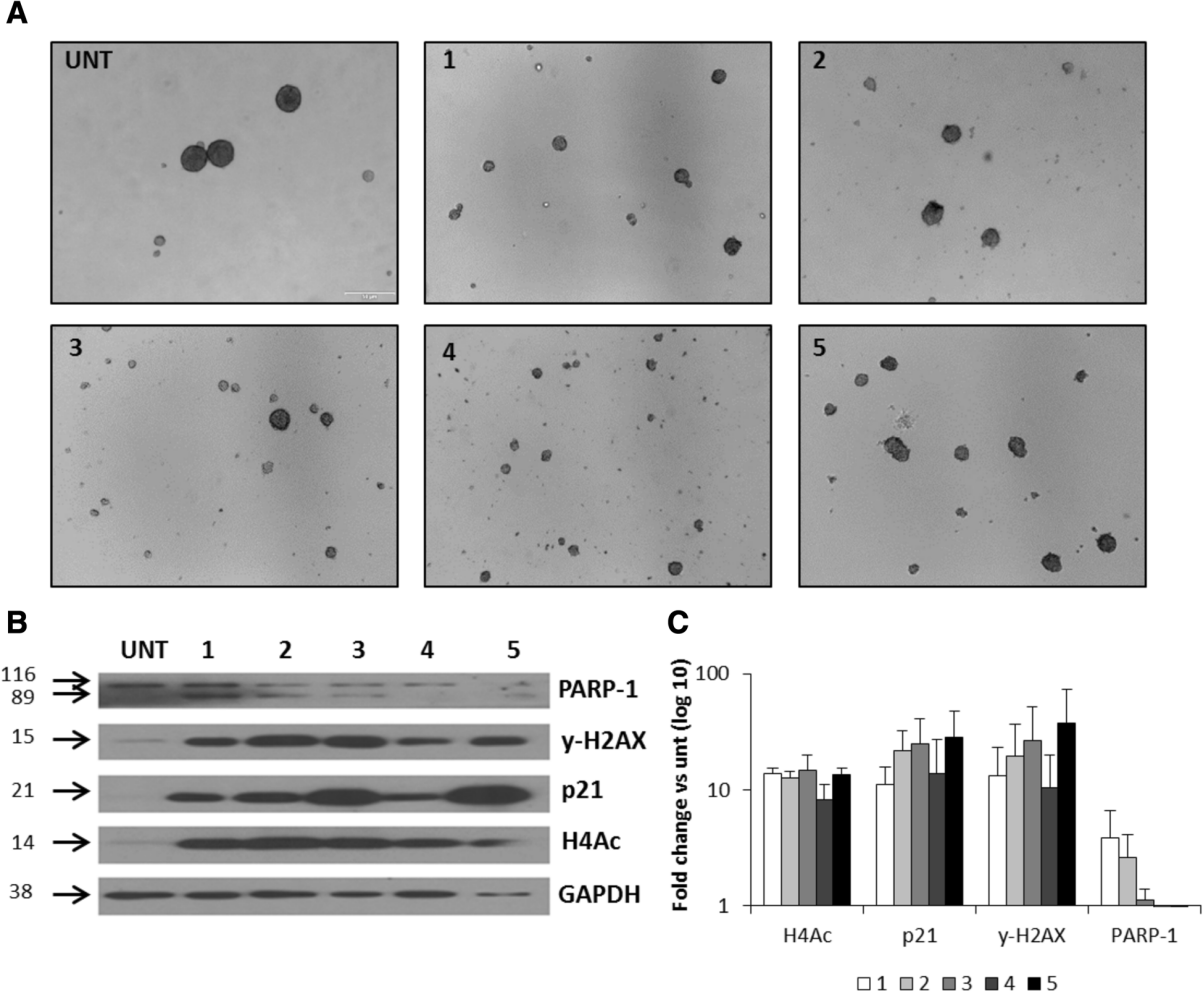
Effects of 1–5 on human glioma CSCs. LN18 cells were grown under sphere forming conditions for 6 days and then inhibitors were added for 24 h. **a** Representative pictures of LN18-derived spheres show sphere disintegration after exposure to 1–5. **b** At the end of the experiments, spheres were collected by centrifugation and total protein extracts were prepared. Representative immunoblots show western blot analysis of PARP-1 cleavage, γ-H2AX, p21, and AcH4 levels in glioma spheres after 24 h exposure to 5 μM 1–3, 5, and 1 μM 4. **c** Densitometry analysis of western blot of PARP-1 cleavage, γ-H2AX, p21, and AcH4 levels in glioma spheres after 24 h exposure to 5 μM 1–3, 5, and 1 μM 4 **(***n* = 2)

As shown in Fig. 7a, compounds 1, 3, and 4 promoted the disintegration of the big spheres, more efficiently than 5. The treatment with 1–5 led to histone H4 hyperacetylation and significant induction of p21^WAF1^ and γ-H2AX proteins, markers of growth arrest, and DDR pathway, respectively, with a slight increase of the cleaved PARP-1 levels, as evidenced by western blot analysis (Fig. 7b, c).

## Discussion

Histone-modifying enzymes that catalyze reversible lysine acetylation are major players in the epigenetic modulation of gene expression and are recognized targets in anticancer therapies [26]. In recent years, several drugs that modulate histone acetylation/deacetylation have been developed and tested. Acetylation of histone and non-histone proteins by HDACs is an important regulatory mechanism implicated in regulation of glioma cell proliferation [27–29]. Alterations in sequence and/or expression of HDAC coding genes may contribute to GBM pathogenesis and progression [2, 8, 9, 30]. In the present study, we explored the public TCGA database to evaluate *HDAC 1–11* expression in glioma of different grades. The expression of most HDACs correlated inversely with malignancy grade (in line with a previous report) [18], and only class I *HDAC 1–3* and class IIa *HDAC 7* gene expression was increased in malignant gliomas.

We focused on HDAC 1 and HDAC 2, and by using siRNA, we knocked down HDAC 1 or HDAC 2 or both in U-87 MG and LN18 glioma cells. This treatment produced significant antiproliferative effects without affecting cell viability validating the rationale of using HDACi to target GBM cells. The early clinical trials with HDAC inhibitors published so far, including vorinostat (SAHA), panobinostat, romidepsin, and valproic acid as HDACi agent, have demonstrated a good tolerability and some modest clinical benefit. However, there is still a question mark about the optimum dose regimen of these agents and about the intratumoral levels achieved. Experience with other HDAC inhibitors, including mocetinostat, in GBM is limited to preclinical studies. Since none of the pan-HDACi evaluated in clinical trials up to date has been approved for the treatment of glioma, we planned to gain insight and dissect the role of the class I HDAC isoforms in glioma by the use of specific inhibitors 1–4, in comparison with the pan-HDACi 5 [11, 16, 28] as a reference. In particular, biochemical assays established that mocetinostat (1) is a HDAC 1/2 inhibitor [11], compound 106 (2) specifically inhibits HDAC 3 [12], and MC1746 (3) and MC2129 (4) are two UBHA inhibitors selective against class I/IIb HDACs (Additional file 5: Table S1). All tested compounds showed HDAC inhibitory activity by inducing histone H4 hyperacetylation in treated cells. While all tested HDACi affected glioma cell proliferation, mocetinostat (1) and MC2129 (4) displayed the strongest cytotoxic and antiproliferative effects, higher than those showed by the reference SAHA (5), and such effects were substantially maintained even 3 days after drug removal (long-lasting changes), so providing support to the usefulness of class I HDAC inhibition in GBM cells. Moreover, since the HDAC 3 selective inhibitor compound 106 (2) was not so effective in all cellular settings of the study, we postulate that the simultaneous inhibition of HDAC 1 and 2 is the best strategy for achieving anticancer effects in glioma cells.

When exposed to HDACi at 1–10 μM concentrations, NHA were relatively more resistant than glioma cells to their cytotoxic effects so suggesting some specificity of these HDACi towards tumor cells.

It is worthy to note that although all compounds induced histone H4 hyperacetylation in both cell lines, different cellular responses to HDACi were observed in LN18 and U-87 MG glioma cells. When exposed to all HDACi, LN18 cells increased the expression of the cell cycle inhibitor p21^WAF1^ that resulted in growth arrest (mostly at the G2/M phase) and non-apoptotic programmed cell death as shown by morphological alterations, upregulated levels of cleaved PARP-1, and by the lack of subG1 subpopulation marking DNA fragmentation. In contrast, U-87 MG cells treated with HDACi mostly exhibited reduced proliferation, with only mocetinostat (1) causing evident G2/M phase arrest, and the appearance of cells with flatten morphology. The differences in cell responses to HDACi could be due to the different molecular background of the two cell lines. In fact, Lee and coworkers showed that the tumor suppressor gene PTEN (phosphatase and tensin homolog), mutated in 36% of GBMs [30, 31], is able to switch the cell fate of glioma cells exposed to ionizing radiation between apoptosis and senescence, with PTEN-proficient LN18 cells that enter apoptosis, while PTEN-deficient U-87 MG cells, with high levels of both AKT activation and intracellular reactive oxygen species (ROS), undergo senescence after exposure to ionizing radiation [32]. Altogether, these results suggest that combining new HDACi with restoration of PTEN activity or using new HDACi in PTEN-proficient glioma cells could represent in the future a powerful anti-glioma strategy.

Finally, we found a potent activity of HDACi, object of the study, against glioma stem cells. There is a growing evidence that CSCs are responsible for cancer initiation, progression, and reduced responses to conventional therapy in many tumors, including GBM [22]. One of the emerging therapeutic approaches is targeting GSCs. Epigenetic enzymes inhibitors, which can induce epigenetic reprogramming, are envisioned as potential anti-tumor therapeutics. HDACi have been shown to affect GSC and enhance the efficacy of temozolomide or radiotherapy [33–35]. Eradication of the stem cell subpopulation is mandatory to achieve an effective treatment for this tumor [36].

We and others have shown previously that sphere cultures expanded from glioma LN18 cells in serum-free medium with growth factors are characterized by the higher expression of pluripotency markers (NANOG, POU5F1, SOX2, CD133) [23–25, 37–41]. LN18 glioma CSC-enriched spheres more closely resembled those derived from primary tumors, in both sphere behavior and self-renewal gene expression in comparison to spheres derived from other cell lines (U-87 MG or GL261 murine cell line) [37]. Furthermore, we have shown that these cells differentiate after serum addition which makes them useful as a glioma CSC model [23]. Mocetinostat (1) and MC2129 (4) induced profound changes in LN18 glioma CSC-enriched spheres: rapid sphere disintegration, DNA damage response with upregulation of phosphoryled H2AX and p21^WAF1^ levels. This observation highlights the capability of new inhibitors to act as effectively on glioma CSCs as on bulk cancer cells. As these cells proliferate more slowly than bulk cells, the differences observed after 24 h treatment in sphere morphology (the size and number of spheres), are rather not associated with growth arrest, but with HDACi toxicity. Nevertheless, we cannot exclude that the treatment with HDACi launch differentiation processes of LN18 glioma CSC-enriched spheres, since it was previously shown that treatment of glioma stem cells with HDACi may lead to differentiation (i.e., SAHA at 5 μM [42]). However, the process of differentiation requires longer time to observe the differences in differentiation markers (7–10 days) and accumulation of additional transcriptional changes that accrue over the course of weeks and months, even after exposure to well-known differentiating agent like BMP4 [43]. Summing up, the presented results are preliminary, but encouraging and providing support for the further study of HDACi tested in in vitro and in vivo preclinical GBM models.

## Conclusions

In conclusion, this study demonstrates the anti-tumor effects in GBM cells of mocetinostat (1) and of the novel uracil-based HDACi MC2129 (4) that are less toxic to normal astrocytes. Both inhibitors induce a cell cycle arrest in the G2/M phase more effectively than the clinically used anticancer HDACi SAHA (5). Moreover, both compounds exert cytotoxic effects on glioma sphere cultures, enriched in CSCs that are highly resistant to conventional therapy, so providing support for their use in vivo in preclinical GBM models.

## Methods

### HDAC inhibitors

HDACi were synthesized and in vitro tested against HDAC 1–11 as described in Additional file 5.

### Cell culture

Human malignant U-87 MG and LN18 glioblastoma cells were purchased from American Type Culture Collection (ATCC). Cells were cultured in Dulbecco’s modified Eagle medium (DMEM) supplemented with 10% fetal bovine serum (Gibco Invitrogen) and 100 units/mL of penicillin, 100 μg/mL of streptomycin, and 0.25 μg/ mL of amphotericin B (Antibiotic-Antimycotic, Thermofisher Scientific). Glioma cells were seeded at a density of 10,000/cm^2^ on the 96 wells (MTT metabolism, BrdU incorporation assays) or 60 mm plates (western blotting, flow cytometry) and left for 24 h. Then, cells were treated with HDACi (dissolved in DMSO) at the indicated concentrations for additional 24 h. HDACi were used in concentration range between 0.5 and 5 μM based on prior evaluation of IC_50_ against selective HDACs (Additional file 5: Table S1). After 24 h of treatment, cells were washed and collected by trypsynization (Gibco Invitrogen) for further analysis.

Normal human astrocytes (NHA) were purchased from Lonza (Walkersville, MD, USA) and cultured in ABM Basal Medium (Lonza) supplemented with 3% fetal bovine serum, 1% l-glutamine, 0.1% ascorbic acid, 0.1% human EGF, 0.1% gentamicin, and 0.0025% recombinant human insulin. All cell cultures were grown in a humidified atmosphere of CO_2_/air (5%/95%) at 37 °C.

### HDAC silencing

Cells were seeded on 6-well (2 × 10^5^) or 96-well plates (5 × 10^3^), and after 24 h, cells were transfected with 25 nM control non-targeting siRNA, and siRNA to HDAC 1 or HDAC 2, or both (ON-TARGET siRNA, Dharmacon) using Viromer Blue transfection reagent (Lipocalyx) at 0.1% dilution in culture medium. After 48 h, the effects of gene silencing on gene and protein expression, cell viability, and proliferation were analyzed.

### TCGA data analysis

TCGA level 3 RNAseq data (aligned by STAR and gene expression counted by HTseq) were uploaded to R. Data from TCGA GBM (glioblastoma) and LGG (lower grade gliomas) repositories were uploaded. Gene expression levels as FPKM (fragments per kilobase of exon per million) were extracted for all genes from the HDAC gene family. Visualization of HDAC expression differences between grades was done in R.

### Real-time PCR

Total RNA was isolated using High Pure RNA Isolation Kit (Roche) and used as a template to synthesize cDNA by extension of oligo(dT)_15_ primers (2.5 mmol/L) with 200 units of SuperScript III Reverse transcriptase (Invitrogen). Real-time PCR amplifications were performed in duplicates in a 10-μL reaction volume containing 1× Fast SYBR Green MasterMix (Applied Biosystems) and a set of primers. Sequences of primer used were as follows: GAPDH-F: TCCTGGAACAGCAAAACAAG; R: CAGCCTCAGGTTGGTTTCAT; HDAC 1: F: CCGAGACGGGATTGATGACG; R: ACACTGTAAGACCACCGCAC; HDAC 2: TCAGTTGCTGGAGCTGTGAAG; R: AGCATGATGTAATCCTCCAGCC. The amount of target mRNA was first normalized to the expression level of the GAPDH gene amplified from the same sample and then to untreated controls. Data were analyzed by the relative quantification (^ΔΔ^Ct) method using Quant Studio 12 K Flex Real-Time PCR System and software (Applied Biosystems).

### Protein isolation, electrophoresis, and detection

Whole cell lysates were prepared by scraping the cells into the buffer containing phosphatase and protease inhibitors (20 mM Tris HCl, pH 6.8, 137 mM sodium chloride, 25 mM β-glycerophosphate, 2 mM sodium pyrophosphate, 2 mM EDTA, 1 mM sodium orthovanadate, 1% Triton X-100, 10% glycerol, 5 μg/ml leupeptin, 5 μg/ml aprotinin, 2 mM benzamidine, 0.5 mM DTT, 1 mM PMSF). The protein concentration was determined with the Pierce BCA Protein Assay Kit (Thermo Scientific). Protein extracts were separated on SDS-PAGE before electrophoretic transfer onto a nitrocellulose membrane (Amersham Biosciences, Germany) as described [44]. After blocking with 5% non-fat milk in TBS-T (Tris-buffered saline pH 7.6/0.15% Tween 20), the membranes were incubated with primary antibodies diluted in a TBS-T overnight at 4 °C and then with relevant secondary antibodies for 1 h at RT. Antibodies recognizing HDAC 1 (#06-720 diluted 1:1000), HDAC 2 (#05-814 diluted 1:1000), acetylated histone H3 (#06-599 diluted 1:10000), and acetylated histone H4 (#06-866 diluted 1:5000) were all purchased from Merck Millipore. P21CIP1 (diluted 1:500, from Santa Cruz, # sc-397), PARP-1 (diluted 1:500, from Cell Signaling, # 9542), γ-H2AX (diluted 1:1000, from Abcam, # ab26350), horseradish peroxidase-conjugated anti-rabbit IgG (#PI-1000 diluted 1:10000), and horseradish peroxidase-conjugated anti-mouse IgG (#PI-2000 diluted 1:10000) were obtained from Vector Laboratories. Immunocomplexes were visualized by using SuperSignal West Pico PLUS Chemiluminescent Substrate (Thermofisher Scientific). Horseradish peroxidase-conjugated anti-β-actin antibody (diluted 1:30000, from Sigma-Aldrich, Saint Louis, MO, USA) or anti-GAPDH antibody (diluted 1:30,000 from Milipore, # MAB374) was used as a loading control. The molecular weight of proteins was estimated with pre-stained protein markers (Sigma-Aldrich, Saint Louis, MO, USA). Densitometry analysis was performed using ImageJ software.

### Cell viability assayed by MTT metabolism

Cell viability was determined by measuring the conversion of MTT (3-(4,5-dimethylthiazol-2-yl)-2,5-diphenyltetrazolium bromide, final concentration of 0.5 mg/mL) to formazan in living cells. U-87 MG and LN18 glioma cells were seeded in 96-well plates at the density of 3 × 10^3^ per well. NHA were plated (4 × 10^4^ of cells per well) on 24-well culture plates coated with 25 μg/ml poly-l-lysine. Then, the cells were cultured in the presence of HDACi for 24 h. After 2–4 h of incubation with MTT at 37^o^ C, formazan crystals were dissolved in a lysis buffer containing 50 mM HCl in isopropanol. Optical density was measured at 570 nm using a scanning multi-well spectrophotometer. MTT and all other reagents were purchased from Sigma Aldrich.

### Measurement of cell proliferation

Proliferation of glioma U-87 MG and LN18 cells was measured in cells cultured in 96-well plates (seeded at the density of 3 × 10^3^ per well) using BrdU incorporation assay (Roche). LN18 or U87 cells were cultured in the presence of HDACi for 24 or 48 h after gene silencing. Then, BrdU was added for 2 h and BrdU incorporation was measured according to the vendor’s protocol.

### DNA content analysis

For DNA analysis, cells were collected by trypsinization, fixed in 70% ethanol, and stained with propidium iodide (PI) solution (3.8 mM sodium citrate, 50 mg/ml RNAse A, 500 mg/ml PI, in PBS). DNA content analyses were performed using a Becton-Dickinson FACS Calibur and the BD CellQuest Pro 6.0 software. At least 10,000 events were analyzed for each sample.

### Sphere cultures and treatment

For sphere-forming assay, cells were seeded at a low density (1500 viable cells/cm^2^) onto non-adherent plates and cultured in DMEM/F-12 medium, supplemented with 2% B27 (Gibco Invitrogen), 20 ng/ml rhbFGF (Miltenyi Biotec), 20 ng/ml rhEGF, and 0.0002% heparin (StemCell Technologies), Antibiotic-Antimycotic (Thermofisher Scientific). Cells were fed every 4 days by adding 1 ml of the fresh medium. After 6 days of culturing, HDACi were added to the sphere cultures for 24 h. Spheres were then collected by centrifugation for further analyses.

### Statistical analysis

Analysis of HDAC 1–11 expression in gliomas from the TCGA repository was performed using ANOVA test with no assumption of equal variance (Welch one-way test) followed by FDR corrections for multiple testing.

All experiments were performed in duplicates or triplicates and repeated four times. Numerical results are expressed with mean values ± standard error of the mean. The respective *p* values were calculated using type 2 two-tailed *t* test (followed by FDR corrections for multiple hypothesis testing), and *p* < 0.05 was considered statistically significant.

## Additional files

Additional file 1: **Figure S1.** (A) Densitometry analysis of western blot shows efficacy of HDAC 1 and HDAC 2 knockdown at protein level in U-87 MG and LN18 cells after gene silencing using specific siRNAs (*n* = 3). The respective *p* values were calculated using type 2 two-tailed *t* test, and *p* < 0.05 was considered statistically significant. *p value < 0.05, **p value < 0.01. (B) Densitometry analysis of western blot for acetylated histones H3 and H4 (H3Ac, H4Ac) in HDAC 1 and HDAC 2 depleted U-87MG and LN18 cells 48 h after siRNA transfection (*n* = 2). (PDF 35 kb)

Additional file 2: **Figure S2.** Effects of 1–5 on normal human astrocytes viability. (A) MTT test for cell viability after exposure to HDACi at indicated concentrations for 24 h. The respective *p* values were calculated using type-2 two-tailed *t* test followed by FDR corrections for multipole hypothesis testing, and *p* < 0.05 was considered statistically significant *p value < 0.05, **p value < 0.01. (B) Morphological effects on normal human astrocytes after exposure to 5 μM 1–3, 5, and 1 μM 4 for 24 h. (PDF 422 kb)

Additional file 3: **Figure S3.** Pictures showing morphological changes of U-87 MG and LN18 cells after exposure to 5 μM 1–3, 5, and 1 μM 4 for 24 h. (PDF 841 kb)

Additional file 4: **Figure S4.** Pictures showing long-term effects of 1–5 on U-87 MG and LN18 cell morphology after exposure to 5 μM 1–3, 5, and 1 μM 4 for 24 h followed by 72 h of cell culture in a HDACi-free medium. (PDF 841 kb)

Additional file 5: **Tables S1.** Supplemental Information. (DOCX 51 kb)

## Abbreviations

BrdU: Bromodeoxyuridine
CSC: Cancer stem cell
DDR: DNA damage response
GBM: Glioblastoma
HDAC: Histone deacetylase
HDACi: Histone deacetylase inhibitor
LGG: Low-grade glioma
MTT: 3-(4,5-Dimethylthiazol-2-yl)-2,5-diphenyltetrazolium bromide
NHA: Normal human astrocytes
PARP-1: Poly(ADP-ribose) polymerase 1
PTEN: Phosphatase and tensin homolog
SAHA: Suberoylanilide hydroxamic acid
siRNA: Short interfering RNA
TCGA: The Cancer Genome Atlas
UBHA: Uracil-based hydroxyamide
WHO: World Health Organization
