# Supplementary figures and images for: Histone deacetylase inhibitors exert anti-tumor effects on human adherent and stem-like glioma cells
Source: Clin Epigenetics. 2019 Jan 17;11:11. doi: 10.1186/s13148-018-0598-5 (PMC6337817; doi:10.1186/s13148-018-0598-5)

**A**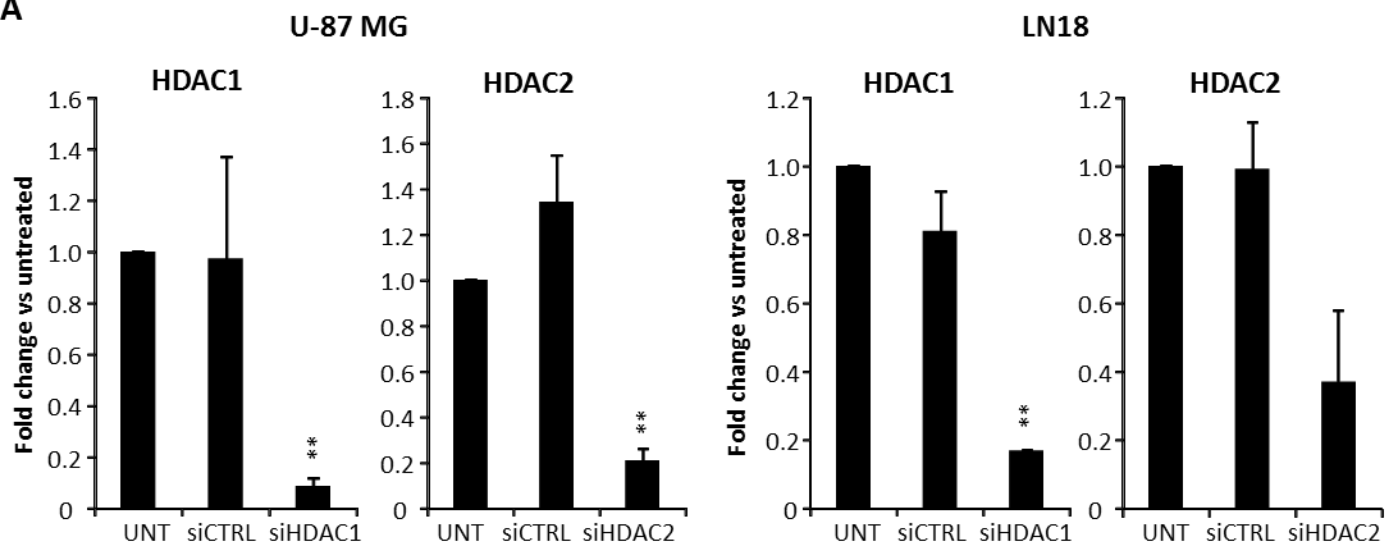**B**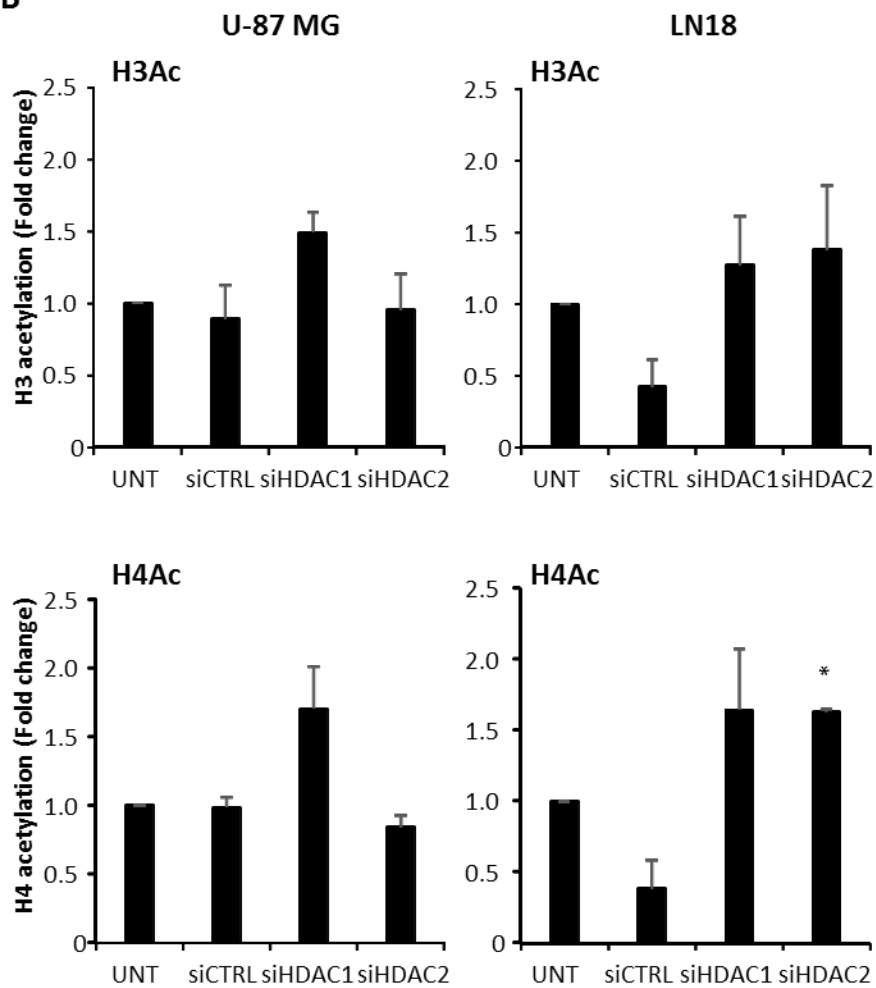

Supplement: Supplementary file 1 — Figure S1. (A) Densitometry analysis of western blot shows efficacy of HDAC 1 and HDAC 2 knockdown at protein level in U-87 MG and LN18 cells after gene silencing using specific siRNAs (n = 3). The respective p values were calculated using type 2 two-tailed t test, and p < 0.05 was considered statistically significant. *p value < 0.05, **p value < 0.01. (B) Densitometry analysis of western blot for acetylated histones H3 and H4 (H3Ac, H4Ac) in HDAC 1 and HDAC 2 depleted U-87MG and LN18 cells 48 h after siRNA transfection (n = 2). (PDF 35 kb) [file 13148_2018_598_MOESM1_ESM.pdf]

**A**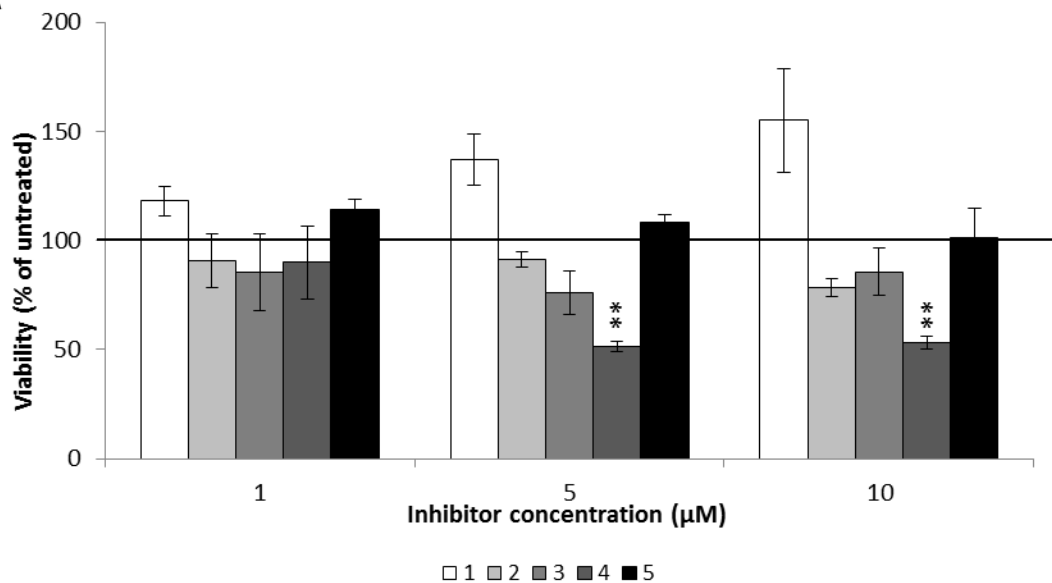**B**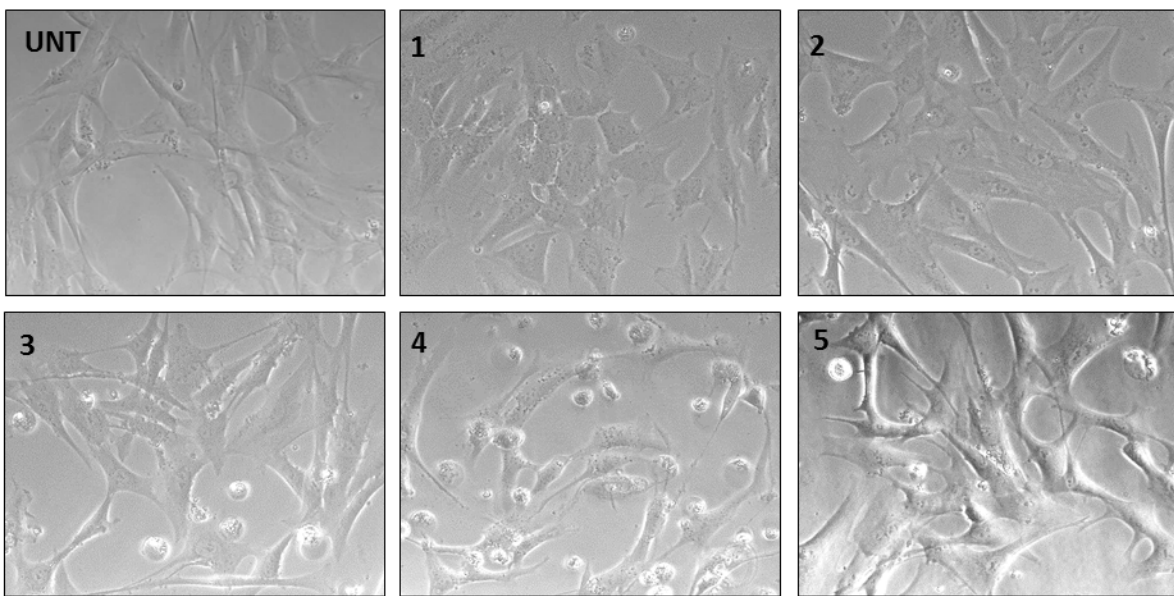

Supplement: Supplementary file 2 — Figure S2. Effects of 1–5 on normal human astrocytes viability. (A) MTT test for cell viability after exposure to HDACi at indicated concentrations for 24 h. The respective p values were calculated using type-2 two-tailed t test followed by FDR corrections for multipole hypothesis testing, and p < 0.05 was considered statistically significant *p value < 0.05, **p value < 0.01. (B) Morphological effects on normal human astrocytes after exposure to 5 μM 1–3, 5, and 1 μM 4 for 24 h. (PDF 422 kb) [file 13148_2018_598_MOESM2_ESM.pdf]

## U-87 MG

UNT

1

2

3

4

5

## LN18

UNT

1

2

3

4

5

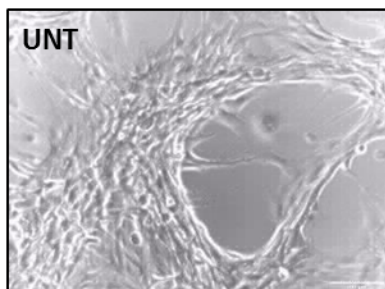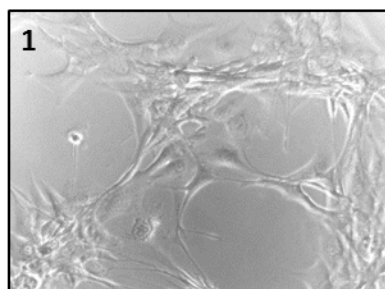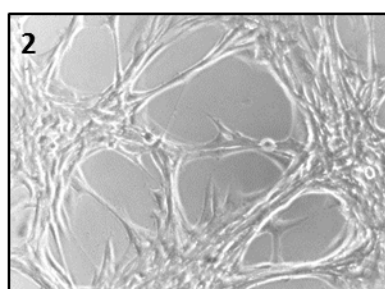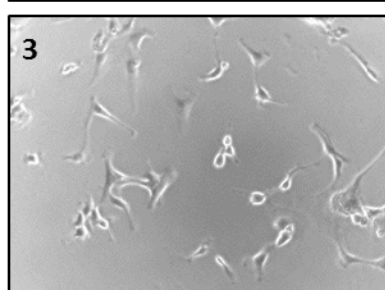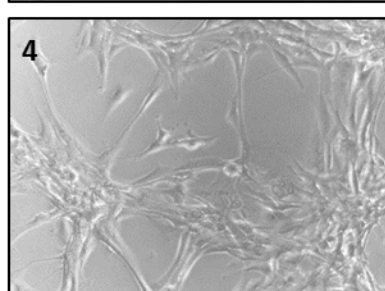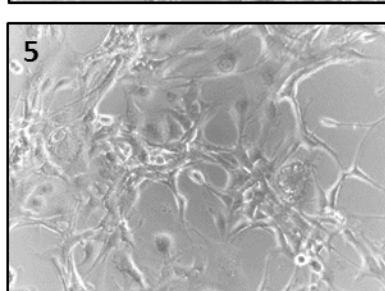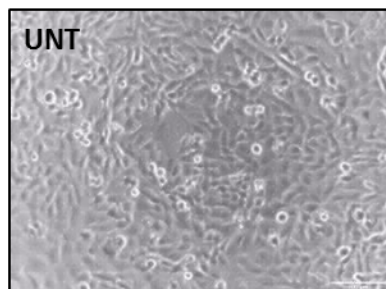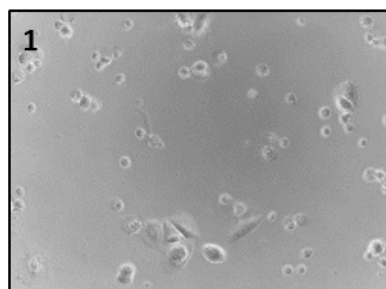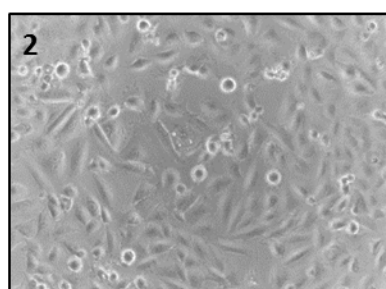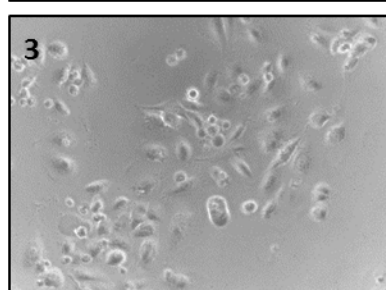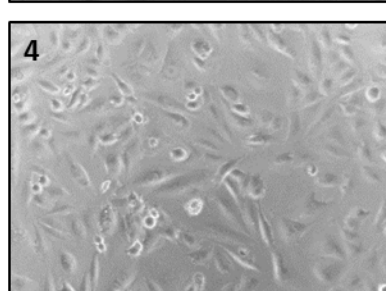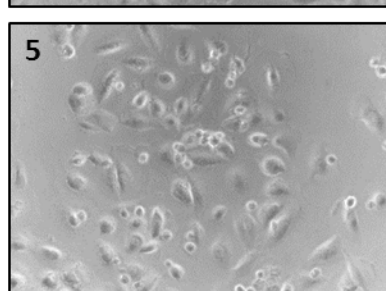

Supplement: Supplementary file 3 — Figure S3. Pictures showing morphological changes of U-87 MG and LN18 cells after exposure to 5 μM 1–3, 5, and 1 μM 4 for 24 h. (PDF 841 kb) [file 13148_2018_598_MOESM3_ESM.pdf]

## U-87 MG

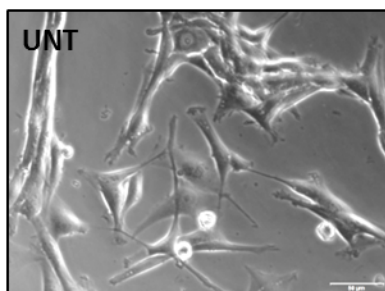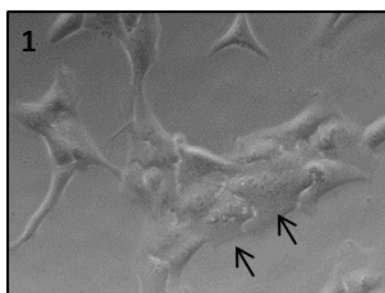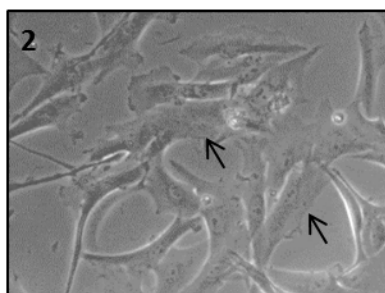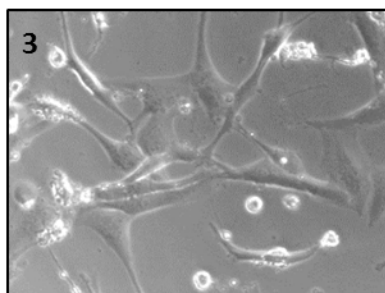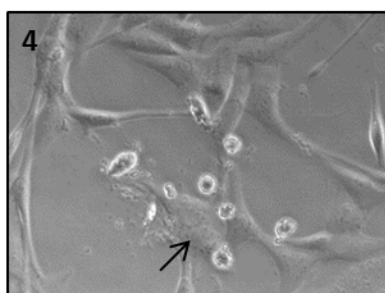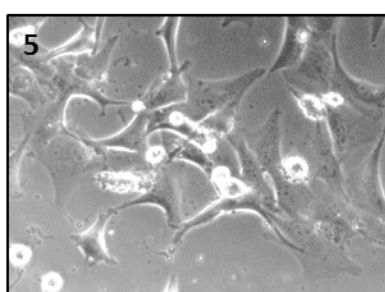

## LN18

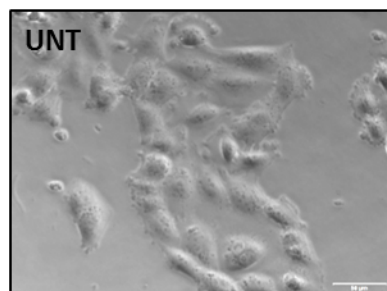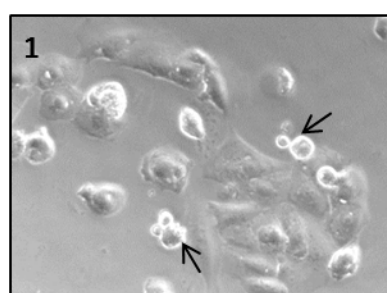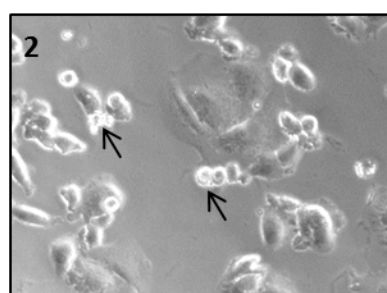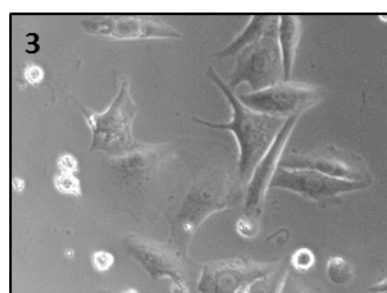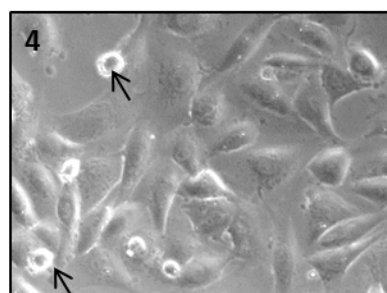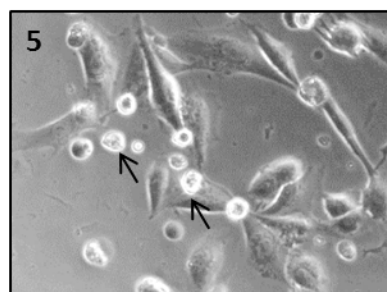

Supplement: Supplementary file 4 — Figure S4. Pictures showing long-term effects of 1–5 on U-87 MG and LN18 cell morphology after exposure to 5 μM 1–3, 5, and 1 μM 4 for 24 h followed by 72 h of cell culture in a HDACi-free medium. (PDF 841 kb) [file 13148_2018_598_MOESM4_ESM.pdf]
