# Supplementary material for: Histone deacetylase inhibitors exert anti-tumor effects on human adherent and stem-like glioma cells
Source: Clin Epigenetics. 2019 Jan 17;11:11. doi: 10.1186/s13148-018-0598-5 (PMC6337817; doi:10.1186/s13148-018-0598-5)
Supplement: Supplementary file 5 — Tables S1. Supplemental Information. (DOCX 51 kb) [file 13148_2018_598_MOESM5_ESM.docx]

**Histone Deacetylase Inhibitors Exert Anti-Tumor Effects on Human Adherent and Stem-like Glioma Cells**

Halina Was, Sylwia K. Krol, Dante Rotili, Antonello Mai, Bartosz Wojtas, Bozena Kaminska, Marta Maleszewska

**Supplemental Information**

**Results**

*Chemistry*

Compounds **1**, [1] **2** [2] and **5** [3] were prepared as reported in literature. The synthesis of compounds **3** and **4** is depicted in Scheme 1. The known 2-mercapto-6-(naphthalen-2-yl)pyrimidin-4(3*H*)-one **6** [4] was treated with the commercially available ethyl 6-bromohexanoate in the presence of anhydrous potassium carbonate to afford the ethyl ester **7**, which was hydrolyzed to the related carboxylic acid **8** with potassium hydroxide in ethanolic solution at room temperature (rt) (Scheme 1A). The 4-chloro ester **10** was obtained by reaction of compound **9**, previously reported by us [5], with the phosphorus oxychloride-DMF complex under Vilsmeier-Haack conditions. It was then converted to the corresponding carboxylic acid **11** by alkaline hydrolysis with lithium hydroxide in a mixture water/THF at rt (Scheme 1B). The hydroxamates **3** and **4** were finally synthesized from the corresponding carboxylic acids **8** and **11**, respectively, by treatment in sequence with i) ethyl chloroformate and triethylamine in dry THF, ii) *O*-(2-methoxy-2-propyl)hydroxylamine in dry THF, and iii) Amberlyst 15 ion-exchange resin in methanol at rt (Scheme 1A and 1B).


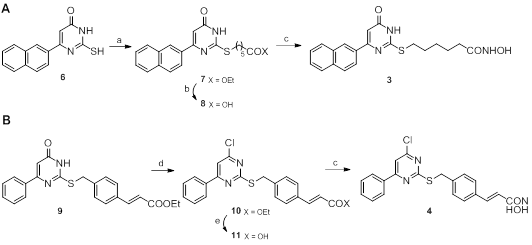


Scheme 1. (a) Ethyl 6-bromohexanoate, anhydrous potassium carbonate, dry DMF, rt; (b) 2N potassium hydroxide, ethanol, rt; (c) (i) ethyl chloroformate, triethylamine, dry THF, 0°C → rt; (ii) *O*-(2-methoxy-2-propyl)hydroxylamine, 0°C → rt; (iii) Amberlyst 15, methanol, rt; (d) dry DMF-phosphorus oxychloride rt, then dry chloroform 9^15^ solution, rt; (e) lithium hydroxide hydrate, THF:water (1:1 v/v), rt.

*Biochemistry*

The two new HDACi **3** and **4** were assayed against all HDAC1-11 isoforms in 10-dose IC_50_ mode with 3-fold serial dilution starting from 50 μM (Table 1). From these data, **3** and **4** emerged as class I/IIb-selective HDACi, being potent at (sub)micromolar level against class I HDACs (HDAC1-3, 8) and class IIb HDACs (HDAC6, 10), while against class IIa HDACs (HDAC4, 5, 7, 9) **3** and **4** did not show inhibition up to 50 μM, the maximum tested dose.

**Table S1.** IC_50_ Values (μM) of **3** and **4** against the HDAC1-11 Isoforms

| HDAC | IC_50_, µM | |
| --- | --- | --- |
|  | **3** | **4** |
| 1 | 0.65 | 1.68 |
| 2 | 0.11 | 1.11 |
| 3 | 0.063 | 0.53 |
| 4 | >50 | >50 |
| 5 | >50 | >50 |
| 6 | 0.01 | 0.12 |
| 7 | >50 | >50 |
| 8 | 0.61 | 2.22 |
| 9 | >50 | >50 |
| 10 | 0.04 | 2.8 |
| 11 | 4.05 | 5.31 |

*Experimental Procedures*

Melting points were determined on a Buchi 530 melting point apparatus and are uncorrected. ^1^H-NMR spectra were recorded at 400 MHz on a Bruker AC 400 spectrometer; reporting chemical shifts in δ (ppm) units relative to the internal reference tetramethylsilane. All compounds were routinely checked by TLC and ^1^H-NMR. TLC was performed on aluminum-backed silica gel plates (Merck DC, Alufolien Kieselgel 60 F_254_) with spots visualized by UV light. Yields of all reactions refer to the purified products. All chemicals were purchased from Aldrich Chimica, Milan (Italy), and were of the highest purity. Mass spectra were recorded on a API-TOF Mariner by Perspective Biosystem (Stratford, Texas, USA), samples were injected by an Harvard pump using a flow rate of 5-10 µL/min, infused in the Electrospray system. Elemental analyses of the hydroxamic acids were obtained by a PE 2400 (Perkin- Elmer) analyzer and have been used to determine purity of the described compounds that is > 95%. Analytical results are within ± 0.40% of the theoretical values.

*General procedure for the preparation of hydroxamates* ***3*** *and* ***4***

To a 0 °C cooled solution of the proper carboxylic acid **8** or **11** (0.87 mmol, 1 equiv) in dry THF (8 mL), triethylamine (2.3 mmol, 0.32 mL, 2.6 equiv) and ethyl chloroformate (2.1 mmol, 0.2 mL, 2.4 equiv) were added in sequence and the resulting mixture was stirred at rt for 10min. The white salt was filtered off, and to the filtrate was added *O*-(2-methoxy-2-propyl)hydroxylamine (5.2 mmol, 0.39 mL, 6 equiv). The resulting mixture was stirred at rt for 1-3h, then was evaporated under reduced pressure and the residue diluted with methanol (3.0 mL). Amberlyst 15 ion-exchange resin (175 mg) was added to the solution of the *O*-protected hydroxamate, and the mixture was stirred at rt for 1-2h. Afterward, the reaction was filtered and the filtrate was concentrated in vacuum to give the crude hydroxamates **3** or **4**, which were finally purified by crystallization from the proper solvents.

*N-hydroxy-6-((4-(naphthalen-2-yl)-6-oxo-1,6-dihydropyrimidin-2-yl)thio)hexanamide (****3****)*: crystallization solvent:acetonitrile; mp: 144-146°C; yield: 57%. ^1^H-NMR (DMSO-*d*_6_) δ 1.44 (m, 2H, CH_2_CH_2_C*H*_2_CH_2_CH_2_S), 1.57 (m, 2H, CH_2_C*H*_2_CH_2_CH_2_CH_2_S), 1.77 (m, 2H, (m, 2H, CH_2_CH_2_CH_2_C*H*_2_CH_2_S), 1.97 (t, 2H, C*H*_2_CONHOH), 3.28 (t, 2H, C*H*_2_S), 6.82 (s, 1H, C5-H), 7.57 (m, 2H, naphthalene ring), 7.94-8.15 (m, 4H, naphthalene ring), 8.68 (m, 2H, naphthalene ring and CON*H*OH), 10.36 (s, 1H, CONHO*H*), 12.75 (s, 1H, uracil N*H*). ^13^C-NMR (DMSO-*d*_6_) δ 23.8, 24.6, 26.2, 31.3, 32.8, 110.9, 123.0, 125.2, 126.4, 126.0, 127.8, 128.1, 128.5, 133.1, 133.8, 134.2, 156.1, 160.8, 166.5, 171.1. Anal. (C_20_H_21_N_3_O_3_S) % Calcd: C, 62.64; H, 5.52; N, 10.96; S, 8.36. Found (%): C, 62.81; H, 5.57; N, 10.82; S, 8.26. MS (ESI), m/z: 384 [M + H]^+^.

*3-(4-(((4-Chloro-6-phenylpyrimidin-2-yl)thio)methyl)phenyl)-N-hydroxyacrylamide (****4****)*: crystallization solvent: methanol; mp:180-182 °C; yield:62%. ^1^H-NMR (DMSO-*d*_6_) δ 4.53 (s, 2H, SC*H*_2_), 6.42 (d, 1H, CH=C*H*CONHOH), 7.41 (d, 1H, C*H*=CHCONHOH), 7.50-7.60 (m, 7H, benzene rings), 8.00 (s, 1H, C5-H), 8.21 (d, 2H, benzene ring), 9.05 (s, 1H, CON*H*OH), 10.73 (s, 1H, CONHO*H*). ^13^C-NMR (DMSO-*d*_6_) δ 34.5, 116.0, 118.9, 127.4 (2C), 127.6 (2C), 128.5 (2C), 128.9, 129.3 (2C), 133.8, 135.7, 136.3, 141.5, 161.2, 161.8, 165.3, 172.5. Anal. (C_20_H_16_ClN_3_O_2_S) % Calcd: C, 60.37; H, 4.05; Cl, 8.91; N, 10.56; S, 8.06. Found (%): C, 60.56 H, 4.09; Cl, 8.99; N, 10.45; S, 7.97. MS (ESI), m/z: 398 [M + H]^+^.

*Preparation of Ethyl 6-((4-(Naphthalen-2-yl)-6-oxo-1,6-dihydropyrimidin-2-yl)thio)hexanoate* **(7).** A mixture of 2-mercapto-6-(naphthalen-2-yl)pyrimidin-4(3*H*)-one **6** [4] (3.94 mmol, 1000 mg, 1 equiv), ethyl 6-bromohexanoate (4.33 mmol, 0.77 mL, 1.1 equiv), and anhydrous potassium carbonate (4.33 mmol, 598 mg, 1.1 equiv) in 3 mL of dry DMF was stirred at rt for 1 h. After quenching with cold water (60 mL), the resulting white precipitate in suspension was filtered and washed over the filter with cold water to provide the crude **7** which was purified by crystallization from acetonitrile-methanol; mp:128-130 °C; yield: 56%. ^1^H-NMR (CDCl_3_) δ 1.23 (t, 3H, COOCH_2_C*H*_3_), 1.59 (m, 2H, SCH_2_CH_2_C*H*_2_CH_2_CH_2_CO), 1.73 (m, 2H, SCH_2_CH_2_CH_2_C*H*_2_CH_2_CO), 1.91 (m, 2H, SCH_2_C*H*_2_CH_2_CH_2_CH_2_CO), 2.34 (t, 2H, CH_2_COO), 3.40 (t, 2H, CH_2_S), 4.11 (q, 2H, COOC*H*_2_CH_3_), 6.83 (s, 1H, C5-H), 7.56 (m, 2H, naphthalene ring), 7.88-8.02 (m, 4H, naphthalene ring), 8.55 (m, 1H, naphthalene ring), 13.45 (br s, 1H, NH). MS (ESI), m/z: 397 [M + H]+.

*Preparation of 6-((4-(Naphthalen-2-yl)-6-oxo-1,6-dihydropyrimidin-2-yl)thio)hexanoic acid* **(8).** A mixture of **7** (1.7 mmol, 660 mg, 1 equiv), 2 N potassium hydroxide (6.6 mL, 7.8 equiv) and ethanol (10 mL) was stirred at rt for 18 h. The solution was poured into water (40 mL) and extracted with ethyl acetate (2 × 25 mL). 2 N Hydrochloric acid was added to the aqueous layer until the pH was 2 and the precipitate was filtered and crystallized from acetonitrile to yield the title compound **8** as a TLC pure white solid; mp: 174-176 °C; yield: 90%. ^1^H-NMR (DMSO-*d*_6_) δ 1.43 (m, 2H, CH_2_CH_2_C*H*_2_CH_2_CH_2_S), 1.58 (m, 2H, CH_2_C*H*_2_CH_2_CH_2_CH_2_S), 1.76 (m, 2H, (m, 2H, CH_2_CH_2_CH_2_C*H*_2_CH_2_S), 1.96 (t, 2H, CH_2_CONHOH), 3.25 (t, 2H, CH_2_S), 6.79 (s, 1H, C5-H), 7.56 (m, 2H, naphthalene ring), 7.96-8.18 (m, 4H, naphthalene ring), 8.66 (m, 1H, naphthalene ring), 12.65 (s, 1H, uracil NH). MS (ESI), m/z: 367 [M - H].

*Preparation of Ethyl 3-(4-(((4-Chloro-6-phenylpyrimidin-2-yl)thio)methyl)phenyl)acrylate* **(10).** A mixture of dry DMF (0.65 mL, 8.47 mmol, 1.75 equiv) and phosphorus oxychloride (0.79 mL, 8.47 mmol, 1.75 equiv) was stirred at rt for 1 h; then a solution of **9** [5] (1.90 g, 4.84 mmol, 1 equiv) in dry chloroform (23 mL) was added. The resulting mixture was stirred at rt for 2 h; then the reaction was quenched with saturated aqueous sodium hydrogen carbonate (80 mL) and the phases were separated. The aqueous layer was extracted twice with fresh chloroform (4 × 80 mL), and the organic extracts were collected, washed with brine, dried, and evaporated to give a residue, which was purified by silica gel column chromatography eluting with a mixture ethyl acetate/hexane (1:10 v/v) to provide pure **10**; mp: 101-103 °C; crystallization solvent: toluene; yield: 75%. ^1^H- NMR (CDCl_3_) δ 1.33 (t, 3H, CH_2_C*H*_3_), 4.25 (q, 2H, OC*H*_2_CH_3_), 4.46 (s, 2H, SCH_2_), 6.40 (d, 1H, CH=C*H*COOEt), 7.37 (s, 1H, C5-H), 7.44-7.52 (m, 7H, benzene rings), 7.65 (d, 1H, C*H*=CHCOOEt), 7.98-8.00 (d, 2H, benzene ring). MS (ESI), m/z: 411 [M + H]+.

*Preparation of 3-(4-(((4-Chloro-6-phenylpyrimidin-2-yl)thio)methyl)phenyl)acrylic Acid* **(11).** A mixture of **10** (500 mg, 1.22 mmol, 1 equiv), lithium hydroxide hydrate (112 mg, 2.68 mmol, 2.2 equiv), THF (4.3 mL) and water (4.3 mL) was stirred at rt for 18 h. The solution was evaporated under reduced pressure and the residue poured into water (50 mL) and extracted with ethyl acetate (2 × 20 mL). 2 N Hydrochloric acid was added to the aqueous layer until the pH was 2 and the resulting white solid in suspension was filtered and purified by silica gel column chromatography eluting with a mixture ethyl acetate/hexane (1:3 v/v) to obtain the desired product as a TLC pure white solid; mp: 200-202 °C; crystallization solvent: acetonitrile; yield: 85%. ^1^H-NMR (CDCl_3_) 4.47 (s, 2H, SCH_2_), 6.40 (d, 1H, CH=C*H*COOH), 7.38 (s, 1H, C5-H), 7.45-7.52 (m, 7H, benzene rings), 7.72 (d, 1H, C*H*=CHCOOH), 7.99 (d, 2H, benzene ring). MS (ESI), m/z: 381 [M - H].

*Biology*

*HDAC1-11 Isoforms Inhibition Assay.*

Individual IC_50_ values for each HDAC isozyme were measured with the homogeneous fluorescence release HDAC assay. Purified recombinant enzymes were incubated with serial diluted inhibitors at the indicated concentration. The deacetylase activities of HDACs 1, 2, 3, 6, and 10 were measured by assaying enzyme activity using AMC-K(Ac)GL substrate and AMC-K(TFA)GL substrate for HDACs 4, 5, 7, 8, 9, and 11 as previously described.^33, 34^ Deacetylated AMC-KGL was sensitive toward lysine peptidase, and free fluorogenic 4-methylcoumarin-7-amide (MCA) was generated, which can be excited at 355 nm and observed at 460 nm. The data was analyzed on a plate to plate basis in relationship to the control and imported into analytical software (GraphPad Prism).

**REFERENCES**

1. Zhou N, Moradei O, Raeppel S, Leit S, Frechette S, Gaudette F, Paquin I, Bernstein N, Bouchain G, Vaisburg A *et al*: **Discovery of N-(2-aminophenyl)-4-[(4-pyridin-3-ylpyrimidin-2-ylamino)methyl]benzamide (MGCD0103), an orally active histone deacetylase inhibitor**. *J Med Chem* 2008, **51**(14):4072-4075.

2. Chou CJ, Herman D, Gottesfeld JM: **Pimelic diphenylamide 106 is a slow, tight-binding inhibitor of class I histone deacetylases**. *J Biol Chem* 2008, **283**(51):35402-35409.

3. Mai A, Esposito M, Sbardella, G., Massa S: **A new facile and expeditious synthesis of N*-*hydroxy-N′-phenyloctanediamide, a potent inducer of terminal cytodifferentiation.** *Org Prep Proc Int* 2001, **33**:391-394.

4. Libermann D, Himbert J, Hengl L: **Antithyroid substances. I. Naphthylthiouracils.** *Bull Soc Chim Fr* 1950:486-489.

5. Mai A, Massa S, Rotili D, Simeoni S, Ragno R, Botta G, Nebbioso A, Miceli M, Altucci L, Brosch G: **Synthesis and biological properties of novel, uracil-containing histone deacetylase inhibitors**. *J Med Chem* 2006, **49**(20):6046-6056.
